# Essential Oil of *Croton zehntneri* Prevents Conduction Alterations Produced by Diabetes Mellitus on Vagus Nerve

**DOI:** 10.3390/plants10050893

**Published:** 2021-04-28

**Authors:** Kerly Shamyra Silva-Alves, Francisco Walber Ferreira-da-Silva, Andrelina Noronha Coelho-de-Souza, José Henrique Leal-Cardoso

**Affiliations:** 1 Laboratory of Electrophysiology, Superior Institute of Biomedical Science, State University of Ceará, Fortaleza CEP 60.740-000, Brazil; shamyrabio@gmail.com (K.S.S.-A.); walberferreira@gmail.com (F.W.F.-d.-S.); andrelinanoronha@gmail.com (A.N.C.-d.-S.); 2 Center of Exact Sciences and Technology, State University of Vale do Acaraú, Sobral CEP 62.042-030, Brazil; 3 Laboratory of Experimental Physiology, Superior Institute of Biomedical Science, State University of Ceará, Fortaleza CEP 60.740-000, Brazil

**Keywords:** *Croton zehntneri*, essential oil, autonomic diabetic neuropathy, vagus nerve, compound action potential

## Abstract

Autonomic diabetic neuropathy (ADN) is a complication of diabetes mellitus (DM), to which there is no specific treatment. In this study, the efficacy of the essential oil of *Croton zehntneri* (EOCz) in preventing ADN was evaluated in the rat vagus nerve. For the two fastest conducting myelinated types of axons of the vagus nerve, the conduction velocities and rheobase decreased, whilst the duration of the components of the compound action potential of these fibers increased. EOCz completely prevented these DM-induced alterations of the vagus nerve. Unmyelinated fibers were not affected. In conclusion, this investigation demonstrated that EOCz is a potential therapeutic agent for the treatment of ADN.

## 1. Introduction

Diabetes mellitus (DM) is a chronic disease that frequently damages the nerves resulting in diabetic neuropathy (DN), which is the most debilitating and prevalent diabetic complication. DN affects approximately two-thirds of diabetic patients [1,2,3]. There is also growing evidence that, even in pre-diabetic conditions, the development of some form of DN occurs [4,5,6,7,8]. DN can affect the nerves of the autonomic nervous system causing autonomic diabetic neuropathy (ADN) that is manifested by the dysfunction of one or more organic systems, the cardiovascular and gastrointestinal systems being the most frequently affected. It is a serious and relatively prevalent complication that is associated with an increased risk of cardiovascular mortality, and with multiple symptoms and damage to the quality of life of diabetic patients [8,9,10].

Despite its serious consequences, DN pathophysiology remains poorly understood and further studies are required to determine more effective treatments. The need for new drugs for DN treatment has been investigated in several studies [11,12,13]. In the search for new treatments for DN, three major components of the physiopathology of the disease are to be stressed: pain, inflammation, and the deterioration of redox balance [14,15,16,17]. Our research team studied the plant *Croton zehntneri* (Euphorbiaceae), abundant in the Brazilian Caatinga, which has widespread use in folk medicine [18,19]. The essential oil of *Croton zehntenri* (EOCz) has pharmacological effects that are likely to be relevant in the treatment of DN, such as: antioxidant and antinociceptive effects, wound healing, and the blockade of neuronal excitability [20,21,22,23,24]. EOCz is composed mainly of the monoterpenoids anethole (80.03%) and estragole (9.53%), which are isomers of eugenol. Its major constituents generally have pharmacological effects similar to those of EOCz [23,24,25], including potent anti-inflammatory activity [26]. Additionally, EOCz has low toxicity documented in sub-chronic administration (dosed 1–300 mg/kg, p.o.) and in acute studies (its LD50 was 3.5 g/kg, oral route), a property which increases the potentiality of this essential oil for use in DN therapy [22,27].

Taken together, several facts related to ADN and EOCz, such as: (i) the disabling and ominous effects of ADN, and the reduced expectancy and quality of life for diabetic patients; (ii) the absence of an effective treatment for ADN; (iii) the pharmacological properties of EOCz; and (iv) the low toxicity of EOCz, led us to hypothesize that EOCz is a good potential therapeutic agent for the treatment of ADN. Thus, the present study was designed to investigate whether EOCz has a protective effect against neural derangements produced by DN. DM-induced alterations in the vagus nerve, which is an important parasympathetic nerve involved in the regulation of various viscera, was selected as an ADN model. EOCz showed a robust neuroprotective effect, which is documented here.

## 2. Results

### 2.1. Streptozotocin-Induced DM Model and EOCz Effects

**Figure 1:**
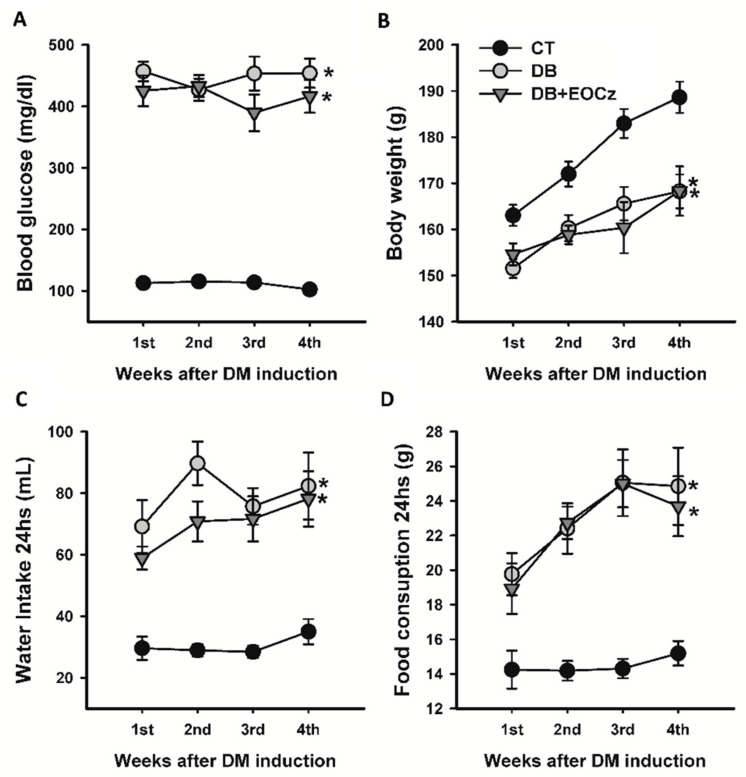
Characterization of the experimental model of diabetes mellitus induced by streptozotocin in adult rats. Panel (**A**) shows post-prandial blood glucose concentration; panel (**B**) shows body mass; panel (**C**) shows water intake; and panel (**D**) shows food consumption. Same legend for all panels. CT, control; DB, diabetic; and DB + EOCz, diabetic treated with EOCz groups. The data are expressed as mean ± E.PM and * *p* < 0.05 compared to all isochronous respective values of the CT group, one-way ANOVA followed by the Dunn’s or Holm–Sidak method. There were no statistical differences between groups DB and DB + EOCz.

Immediately before streptozotocin (STZ) administration, the post-prandial blood glucose concentrations for the control (CT), diabetic (DB), and diabetic treated with EOCz (DB + EOCz) groups were 99.00 ± 3.53, 103.70 ± 3.77, and 103.22 ± 3.76, respectively; these values did not differ significantly (*p* = 0.48; one-way analysis of variance (ANOVA)). After 48 h of STZ administration, the average post-prandial blood glucose concentration of the rats in the DB (*n* = 22) and DB + EOCz (*n* = 18) groups were, respectively, 456.91 ± 15.93 and 425.22 ± 25.14 mg/dL, significantly higher than the animals that received only the citrate solution (CT group = 112.75 ± 3.97 mg/dL; *n* = 22; *p* < 0.001; one-way ANOVA, followed by the Dunn’s method). As shown in Figure 1A, the blood glucose levels remained approximately stable over the four weeks of observation, and the values at the end of this period were 102.38 ± 4.70, 453.86 ± 23.63, and 416.21 ± 26.67, respectively, for the CT, DB, and DB + EOCz groups.

Figure 1B shows the development of animal groups in relation to their body mass. On the day of DM-induction, prior to STZ administration, the body mass values did not differ significantly (*p* = 0.33; one-way ANOVA); for CT, DB, and DB + EOCz, the values were 153.25 ± 2.47, 154.04 ± 1.79, and 157.16 ± 2.17, respectively. On euthanasia day, however, the mass of animals in the CT group (188.64 ± 3.38 g) was significantly higher than the DB (168.24 ± 3.64 g) and DB + EOCZ (168.31 ± 5.35 g) groups (*p* < 0.001; one-way ANOVA, followed by the Holm–Sidak method).

Concerning water and food consumption in 24 h between the groups (Figure 1C,D) during the DM time course, it was observed that, at the end of the first week of DM (week 1), animals that received STZ (DB and DB + EOCz groups) already consumed significantly (*p* < 0.05; two-way ANOVA followed by Holm–Sidak method) more water and food than the CT animals. At the end of euthanasia week (week 4), water and food consumption in 24 h remained increased as compared to CT (*p* < 0.05; one-way ANOVA followed by Holm–Sidak method); for the CT, DB, and DB + EOCz groups, the intake of water was, respectively, 35.00 ± 4.08, 82.33 ± 10.84, and 78.18 ± 9.00 mL, and food consumption was 15.19 ± 0.71, 24.85 ± 2.23, and 23.69 ± 1.73 g.

### 2.2. Characterization of Compound Action Potential (CAP)

**Figure 2:**
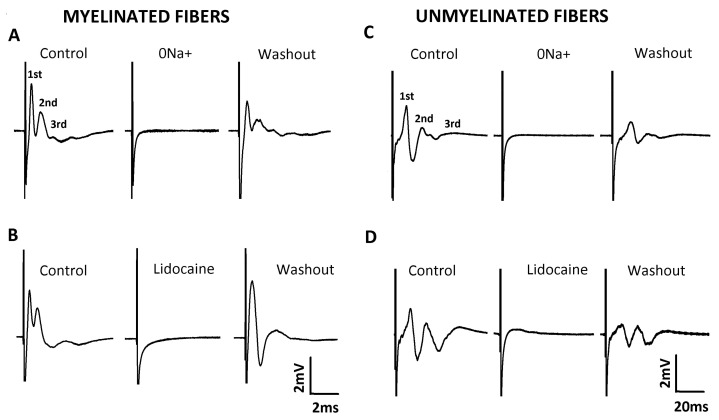
Characterization of extracellular recording of the compound action potential (CAP). Representative traces of the CAP of myelinated (**A**,**B**) and unmyelinated fibers (**C**,**D**). The upper traces illustrate the effect of removing Na^+^ from the extracellular solution, while the lower traces illustrate the effect of lidocaine (1 mM) on the CAP of vagal fibers. The 1st, 2nd, and 3rd indicate the CAP components for both registers.

The technique of extracellular recording of the compound action potential (CAP) was used to investigate the alterations produced by DM on nerve function and the efficacy of EOCz treatment to prevent these alterations. In response to two types of supra-maximal electrical stimuli, two different sets of CAP waves were elicited. Each set presented three waves, which were called 1st, 2nd, and 3rd CAP components, according to the chronological order of appearance in the record (Figure 2). The conduction velocities of the 1st, 2nd, and 3rd components of the CAP evoked by an electrical stimulus of 40 V and 0.1 ms were, respectively, 31.99 ± 2.74, 12.86 ± 1.01 and 5.11 ± 0.39 m/s (*n* = 28). Since all components had the velocity of myelinated fibers, their register was denominated as CAP of myelinated fibers. The components visualized in the CAP evoked by a 60 V and 1.5 ms stimulus had a conduction velocity of 1.51 ± 0.1, 0.66 ± 0.03, and 0.32 ± 0.01 m/s, respectively, and was named CAP of unmyelinated fibers. We also tested if these CAP signals were real or artifactual by means of removing Na^+^ ions from the external nutrient solution (isosmotic substitution by choline chloride) or by applying lidocaine (1mM), a well-known Na^+^ channel blocker, to the nerve. As shown in Figure 2, the removal of Na^+^ ions (panels A and C) or the application of lidocaine (1 mM; panels B and D) abolished the CAP. All these effects were reversible after the restoration of Na+ ions in the external solution or the removal of the lidocaine-containing solution (washout).

### 2.3. DM Effects on CAP of Vagal Fibers and EOCz Prevention

**Figure 3:**
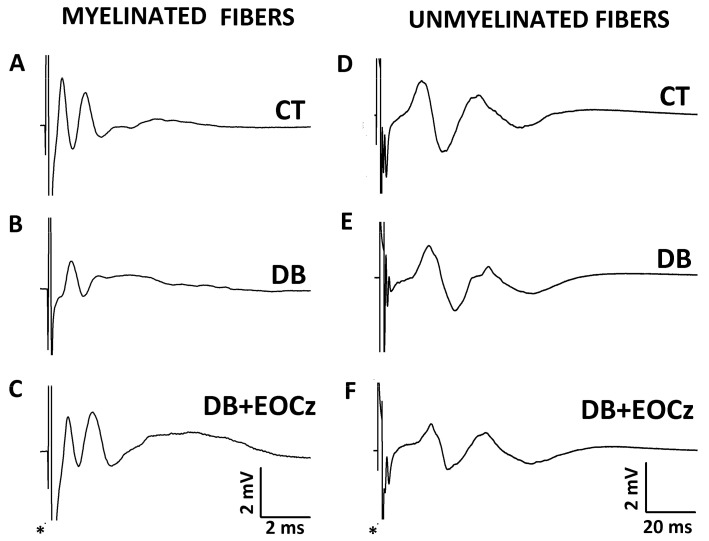
Representative traces of the compound action potential (CAP) of the vagus nerve. The left and right columns show the CAP traces of the myelinated or unmyelinated fibers, respectively, for control (CT, panel **A** or **D**), diabetic (DB, panel **B** or **E**), and diabetic animals treated with EOCz (DB + EOCz, panel **C** or **F**). The asterisks indicate the stimulus artifacts.

**Figure 4:**
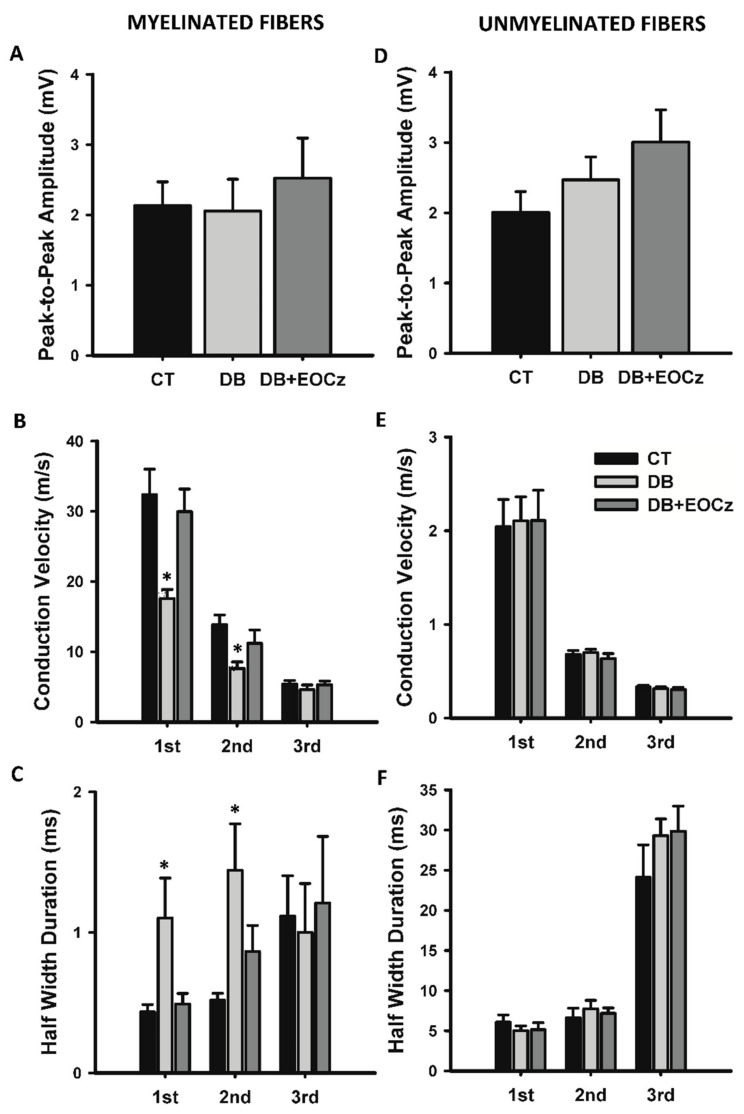
Preventive effect of EOCz treatment on the alterations produced by DM on the conductibility of myelinated and unmyelinated fibers of the vagus nerve. Panels (**A**–**C**) represent peak-to-peak amplitude, conduction velocity and half width duration, respectively, of CAP myelinated fibers, whilst panels (**D**–**F**) represent, respectively, the same above parameters for unmyelinated fibers. The 1st, 2nd, and 3rd indicated at the bottom of each graph refer to the components (waves) viewed on the myelinated and unmyelinated CAP. Same legend for all panels: CT, control; DB, diabetic; DB + EOCz, diabetic treated with EOCz. Data are presented as mean ± E.P.M and *, *p* < 0.05, when compared to control and to DB + EOCz groups (one-way ANOVA, followed by Dunn’s method).

**Figure 5:**
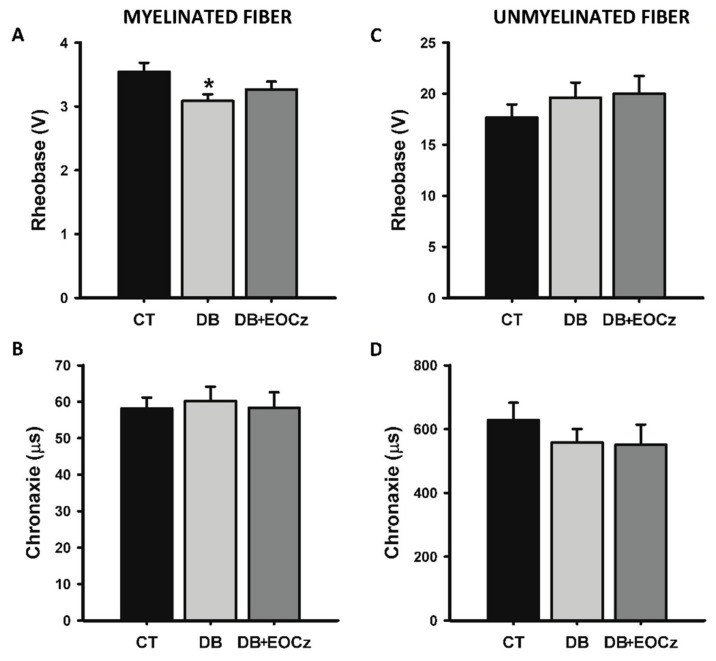
Effect of EOCz treatment on DM-induced alterations of excitability of myelinated and unmyelinated vagal fibers. Rheobase and chronaxie are shown on panels (**A**,**B**), respectively, for myelinated, and on panels (**C**,**D**) for unmyelinated fibers. CT, control; DB, diabetic; DB + EOCz, diabetic treated with EOCz. Data are presented as mean ± E.P.M and * *p* < 0.05, when compared to control (one-way ANOVA, followed by the Holm–Sidak method).

Concerning DM effects on the vagus nerve, as informed by CAP, it affected the conduction velocity, duration, and rheobase of some types of myelinated fibers only, and EOCz treatment prevented these changes (Figure 3, Figure 4 and Figure 5).

As shown in Figure 4, panels A and D, the peak-to-peak amplitude of both fiber groups are not altered. On the other hand, the conduction velocity and duration of the first and second CAP components of the myelinated fibers were affected (Figure 4). The mean values of the conduction velocity of the 1st, 2nd, and 3rd CAP components of the control myelinated fibers were 32.4 ± 3.6, 13.9 ± 1.4, and 5.4 ± 0.5 m/s (*n* = 15), respectively. In the presence of DM (DB group), these values were 17.6 ± 1.3, 7.6 ± 0.9, and 4.6 ± 0.7 m/s (*n* = 17). These values showed significant reductions in conduction velocities for the 1st and 2nd (*p* < 0.05, one-way ANOVA, followed by Dunn’s method), but not for 3rd (*p* = 0.651, one-way ANOVA) CAP components of myelinated fibers as compared to the control. DM treatment with EOCz (300 mg/kg p.o.) was capable of inhibiting the conduction velocity reduction. Mean values of the conduction velocity of EOCz-treated animals were 29.9 ± 3.2, 11.2 ± 1.9, and 5.3 ± 0.6 m/s (*n* = 11) for the 1st, 2nd, and 3rd components; these values did not show statistical difference from the control, but the 1st and 2nd component significantly differed from the corresponding value of the DB group (*p* > 0.05, one-way ANOVA, followed by the Dunn’s method). The conduction velocity of the unmyelinated fibers was not significantly altered by the DM or EOCz treatments (Figure 4).

The induction of DM also altered the duration of the CAP components. As shown in Figure 4C, the duration at 50% of the CAP component amplitude (named half-width duration) was increased significantly in the 1st and 2nd (*p* < 0.05, one-way ANOVA, followed by the Dunn’s method), but not for the 3rd (*p* = 0.950, one-way ANOVA) CAP components of myelinated fibers. Control mean values for the 1st, 2nd, and 3rd CAP components of myelinated fibers were 0.43 ± 0.05, 0.52 ± 0.05, and 1.12 ± 0.29 ms (*n* = 11), respectively. For DM, the values were 1.10 ± 0.28, 1.44 ± 0.33, and 1.00 ± 0.35 ms (*n* = 10). As seen in the conduction velocity, the EOCz treatment was effective in preventing this increase in CAP component durations. The 1st, 2nd, and 3rd CAP components duration for the EOCz treated animals were 0.49 ± 0.07, 0.86 ± 0.19, and 1.21 ± 0.47 ms (*n* = 9), and no statistical significances were found for these values when compared to the control group (*p* > 0.05, one-way ANOVA). For unmyelinated fibers, the CAP components durations were not significantly changed by the DM or EOCz treatments (Figure 4, panel F).

Two CAP parameters specifically related to excitability were investigated: the rheobase and chronaxie. Figure 5 shows the rheobase and chronaxie of myelinated (panel A and B) and unmyelinated (panels C and D) fibers. The rheobase value for the control and DB groups (3.54 ± 0.14 (*n* = 7) and 3.09 ± 0.11 (*n* = 8), respectively) differed significantly (*p* < 0.05, one-way ANOVA, followed by the Holm–Sidak method). Upon the EOCz treatment, rheobase (3.27 ± 0.12 V, *n* = 6) did not differ from control (*p* > 0.05, one-way ANOVA). For the chronaxie of myelinated fibers (Figure 5B) and for the rheobase and chronaxie of unmyelinated fibers (Figure 5C,D), there were no statistical differences between groups and their respective controls (*p* > 0.05, one-way ANOVA).

## 3. Discussion

The major findings of the present study are: (i) DM decreased the excitability and conduction velocity of the two fastest conducting types of myelinated fibers of the vagus nerve and did not affect the other types, including the fibers of C type; and (ii) treatment with EOCz fully prevented DM-induced nerve alterations, but not hyperglycemia. To our knowledge, this is a novel finding.

CAPs are electrically evoked responses measured in the nerve. They provide an insight into the electrophysiology of a population of synchronous firing nerve fibers. Each CAP component corresponds to the activity of a population of fibers with a given average conduction velocity. As such, they offer precise information on average conduction velocities and on the excitability parameters rheobase and chronaxie [28,29]. We collected three CAP components with control velocities corresponding to the activity of myelinated fibers (>2 m/s; Figure 3A) of the types Aβ (32 m/s, 1st component), Aδ (14 m/s, 2nd component), and B (5 m/s, 3rd component). We also collected three components with velocities characteristic of unmyelinated fibers (≤2 m/s) of the vagus nerve (Figure 3D), with the 1st, 2nd, and 3rd showing the velocities 2.0 m/s, 0.7 m/s, and 0.3 m/s, respectively. These data agree with the literature for nerves, including the vagus [30,31,32]. This did not come as surprise, since the vagus nerve is composed of about 25% of myelinated and 75% of unmyelinated fibers in its cervical/thoracic branch [33]. All the six components were abolished by isotonic replacement of Na+ by choline chloride or by 1 mM of the local anesthetic, lidocaine (Figure 2). This demonstrated that the CAPs registered were true representatives of action potential activities and not artifacts. A similar number of CAP components and conduction velocities have been documented for myelinated fibers of the sciatic nerve [6,34,35]. A description of the CAPs of unmyelinated fibers are not easily found in literature. However, Yamasaki, Karakida, and Homma (1990) also described three CAP components for vagal unmyelinated fibers with velocities similar to ours [32].

Since there was an absence of an alteration of the conductibility and excitability of B and C fibers, it is important to mention that the DM adult model that we employed produced a DM with hyperglycemia ([glycose] > 400 mg/dL), a decrease in body weight, polyphagia, and polydipsia. These constitute a full and clear set of characteristics of DM disease as it happens in clinical and experimental situations, as described in the literature [36,37,38]. It is also important to mention that there were nerve alterations, as expected from a disease that is able to induce neuropathy and as reported in the literature for other nerves, like the sciatic [6,35,39]. Thus, we believe that the absence of alterations in the B and C fibers was not due to a lack of true characteristics of DM or to a presence of the light intensity of DM itself, since the signals of severe DM were present, but perhaps a peculiar repercussion of the model or of the short time of evolution of DM (1 month, only). We have no explanation for this lack of significant pathologic activity on B and C fibers. However, we hypothesize that, for the B fibers, the lack of statistical significance for the DM-induced alteration of velocity was due to sample (*n*) size. This is because the arithmetical alteration observed, although not statistically significant, was similar to that observed for other myelinated fibers. Regarding C fibers, besides the short DM duration, our hypothesis is that it might be related to the morphological and functional differences of the interaction of Schwann cells with neural fibers, which largely differs from C to myelinated fibers. It is worth mentioning that, although in clinical studies C fibers are informed to be affected by DM [40,41], some experimental investigations reported no alteration [32,39].

As demonstrated, DM reduced the conduction velocity of the 1st and 2nd components of the CAP of myelinated fibers of the vagus nerve to about 50%. The reduction in the conduction velocity of myelinated fibers by diabetes is widely described in experimental studies, in addition to being considered a gold standard for the diagnosis of diabetic neuropathy [6,35,39,42]. Additionally, DM also promoted an approximately 150% increase in duration of the 1st and 2nd CAP components of vagal myelinated fibers. Ferreira-da-Silva et al. (2013), using another DM model (neonatal, n5-STZ), also observed an increase, although of less magnitude (62%), in the duration of the 2nd component (myelinated fibers of type Aβ) of the sciatic nerves of hyperglycemic animals [6]. We hypothesize that the increase in the duration of the CAP components is probably due to an increase in the desynchronization of the fibers caused by an increased dispersion in the conduction velocity of individual fibers.

Added to the alterations produced in conductibility, DM reduced the rheobase of vagal myelinated fibers. This reduction indicates that these neurons need smaller stimuli to trigger an action potential, that is, they are more easily excitable. The cause of this increase in nervous excitability was not investigated. It may be the result of an increase in sodium currents or changes in the kinetics of membrane Na+ channels, as already documented for sensory neurons in the dorsal root ganglion [6,43,44], or even to a decrease in myelin involutories, as suggested by the decrease in velocity of conduction.

Unlike myelinated, unmyelinated fibers of the vagus nerve did not undergo a DM-induced alteration of CAP parameters. The absence of functional changes in the unmyelinated fibers of the vagus nerve by DM came as a surprise, because these fibers are the smallest in diameter among all fibers and it is widely known that neuropathy is first manifested in small diameter fibers [45]. This study did not investigate the reason for the preferential alteration of myelinated fibers by DM. However, some hypotheses can be discussed. The absence of significant changes in the conduction velocity and duration of vagal unmyelinated fibers may indicate that myelin production by Schwann cells is perhaps more sensitive to the damage caused by DM than another axonal mechanism. In fact, several studies have shown that both the structure and function of Schwann cells are profoundly altered by hyperglycemia [46,47,48,49]. Furthermore, a predominance of demyelinating changes over the axonal degenerations in the abdominal vagus nerve of spontaneously diabetic hamsters has been morphologically demonstrated [50]. Another possible explanation for this apparent resistance is the Remak bundle structure functioning as a physical barrier to toxicity resulting from hyperglycemia [51]. Studies that directly measure the functioning of unmyelinated fibers are scarce in the literature. However, corroborating with our data, Julu (1988) observed in the saphenous nerve of diabetic rats, induced by STZ, that the conduction velocity of myelinated fibers of type Aα is reduced by about 25%, while the velocity of unmyelinated fibers of type C is not altered [39]. Yamasaki, Karakida, and Homma (1990) also observed that the conduction velocities of the unmyelinated components of the vagal CAP were not altered by experimental DM [32].

Oral administration of EOCz (300 mg/kg) for 4 weeks was able to prevent profound alterations in the conductibility and excitability of vagal fibers as demonstrated in this study. To the best of our knowledge, there are few studies in the scientific literature that have investigated the effect of essential oils on DN. In fact, we found studies using an approach similar to ours only for evening primrose oil, which is extracted from the seeds of *Oenothera biennis*. The treatment of STZ-induced rats with this essential oil has improved the sciatic motor and saphenous sensory nerve conduction velocity [52,53,54].

EOCz has been demonstrated to be an active substance on the central (anxiolytic effect) and peripheral (excitability blocker) nervous system [24,55]. Presently, we are demonstrating that it has a neuroprotective effect that occurred, despite the lack of hypoglycemic effect. This fact may represent an interesting advantage in the treatment of DN, since studies have shown that nerve alteration occurs even in a pre-diabetes situation [4,5,6,7,8].

Several studies indicate that inflammatory mechanisms and oxidative stress are important factors in the pathogenesis of DN [14,15,16,17]. Since the neuroprotective effect of EOCz does not depend on glycemic control, its mechanism of action is very likely related to its antioxidant and putative anti-inflammatory effects [20,21]. Although direct demonstration of anti-inflammatory activity has not yet been reported for EOCz, there is indirect evidence for the existence of this effect: (i) Oliveira et al. (2001) suggested that the antinociceptive effect of EOCz could be attributed to an anti-inflammatory effect [22]; (ii) EOCz was also able to heal wounds made on the back of mice after 15 days of treatment, similar to dexamethasone and fibrinolysin [23]; and (iii) EOCz at a dose of 300 mg/kg showed decreased inflammatory cells in the pulmonary parenchyma in the OVA-induced asthma model [21].

EOCz has also been shown to induce the relaxation of several types of smooth muscles so far investigated, such as: tracheal [56], ileum and portal vein [57], and corpora cavernosa [58]. In addition, eugenol, a monoterpenoid similar to anethole, which is the major constituent of EOCz, has been documented to have vasorelaxant activity [59,60]. Taken together, these studies may indicate that EOCz is likely to cause vascular smooth muscle relaxation and improve blood perfusion. Thus, it may represent another mechanism by which EOCz prevents neural alterations.

The neuroprotective effect of EOCz can be attributed to its main constituent, anethole. This constituent of EOCz inhibited early and late cellular responses transduced by the proinflammatory cytokine TNF-α [61], a molecule strongly associated with the genesis of diabetic neuropathy [14], and showed anti-edematogenic activity at relatively low doses (10 mg/kg) [26]. Additionally, anethole had a neuroprotective effect on chronic constriction injury of the sciatic nerve model [62].

In conclusion, we have demonstrated that DM greatly (50%) and unfavorably altered the conduction velocity of myelinated fibers of the vagus nerve but did not affect those unmyelinated. Treatment with EOCz prevented DM-induced alterations. This prevention was not due to glycemia normalization since it occurred in presence of maintained hyperglycemia. Taken together with the low toxicity of EOCz, these facts demonstrate that EOCz is a potential candidate for incorporation into the therapeutic arsenal for the treatment of diabetic neuropathy, likely acting with a mechanism different and complementary to the correction of hyperglycemia.

## 4. Materials and Methods

### 4.1. Plant Extraction and EOCz Analysis and Composition Determination

The extraction method and the composition analysis of the sample of EOCz employed in the present study has been previously published [56]. Briefly, *Croton zehntneri* were collected near the city of Viçosa (lat. 3°33′48″ S.; long. 41°5′41″ W.), CE, Brazil, in the period from June to August 2010. Plant identification was confirmed by botanicals from the State University of Ceará (UECE) and the exsiccate was deposited in the Prisco Bezerra herbarium at the Federal University of Ceará (UFC) and received the protocol number 277477. The leaves were separated from the stems and kept on a surface to dry. Subsequently, the leaves were put in a semi-industrial essential oil extractor (model MA 480, Marconi equipment, Piracicaba, São Paulo, Brazil) that uses a water steam distillation method. The mixture of water and essential oil vapor was collected in a condenser connected to the extractor, the essential oil was separated from aqueous phases, and it was stored in a freezer (approximately −20 °C) in amber glasses until use [56].

The constituents of EOCz were determined by gas-liquid chromatography coupled to mass spectroscopy (model 5971, Hewlett-Packard, Palo Alto, CA, USA) at Parque de Desenvolvimento Tecnológico (PADETEC) of UFC and received the protocol number 002/19.18.2010. The EOCz showed the main following constituents: anethole, estragol (9.53%), bicyclogermacrene (3.98%) and myrcene (1.95%). The other constituents showed individual values inferior to 1.0%, as previously described [56]. The experiments were done between 2011 and 2012.

### 4.2. Animals and DM Induction

This study used the *Rattus norvegicus* species, Wistar rats, of both sexes from the vivarium of the Superior Institute of Biomedical Sciences, State University of Ceará. The animals were kept in an environment with a controlled temperature (24 ± 2 °C) and humidity, 12 h dark/light cycle, and free access to water and food. The handling and use of these animals in all experimental protocols adhered to the rules and directives established by the Ceará State University (UECE) and received approval from the Committee for Ethical Use of Experimental Animals of UECE under the protocol number 12777143-3.

The animals were randomly separated into three groups: control (CT), diabetic (DB), and diabetic treated with EOCz (DB + EOCz). For DM induction, the animals of DB and DB + EOCz groups in the 8th week of life fasted from food for 8 h and received a single injection of streptozotocin (STZ, 65 mg/kg, via i.p.) diluted in sodium citrate buffer (0.1 mM, pH = 4.5). The CT group received only STZ-vehicle solutions. DM was confirmed 48 h after STZ administration by measuring the glycose concentration in caudal blood sample with a glucometer (Accu-Chek^®^, Roche, Mannheim, Germany). For the DB and DB + EOCz groups, only animals which displayed glycose levels superior to 300 mg/dL after 48 h of DM induction were used.

EOCz treatment began after DM confirmation (48 h after STZ administration) and the dose chosen, 300 mg/kg/day of animal body mass, per oral administration (gavage), single daily administration, for 4 weeks, was based on the EOCz efficacy for antinociceptive effects [22]. The DB + EOCz group received the essential oil dissolved in 0.01 % Tween 80 (*v*/*v*) and salina, while the CT and DB groups received vehicle only, following a protocol similar to that of EOCz administration. During this period of treatment, the body mass, glycemic level, and water and food consumption were monitored weekly and the animals were euthanized in the 12th week of life (4 weeks of treatment).

### 4.3. Electrophysiology

For the extracellular recording of the compound action potential (CAP), we followed procedures similar to those previously described [6,24,63,64]. The cervical portion of the vagus nerve was carefully dissected and immediately afterwards immersed in a nutrient solution for at least 30 min for nerve recovery and adaptation to the solution environment. A modified Locke’s solution was used for nerve dissection and tissue nutrition (nutrient solution) in the recording chamber, the composition of which was (in mM): NaCl 140; KCl 5.6; CaCl_2_ 2.2; MgCl_2_ 1.2; and Tris(hydroxymethyl)aminomethane 10, glucose 10. The pH was adjusted to 7.40 ± 0.01 with NaOH/HCl.

The dissected vagus nerve was positioned horizontally on electrodes in a register moist chamber and tissue nutrition was made by immersion of 4–5 mm of nerve in Locke’s solution. For the CAP recording, the proximal end of the vagus nerve was stimulated by a stimulator (model S48, Grass Instruments, Nevada City, CA, USA) coupled to an isolation unit (model SIU 4678, Grass Instruments, Nevada City, CA, USA) with a pre-defined square wave pulse. This stimulation depolarizes vagus nerve fibers, evoking the CAP which propagates through the nerve and can be registered extracellularly in its distal end by a pair of electrodes. The analogic signal was then amplified, visualized in an oscilloscope (model TDS 340A, Tektronix, Beaverton, OR, USA), and converted by an A/D interface (Digidata 1322 A, Axon Instruments, San Jose, CA, USA) to be stored in a computer with Axoscope program (Axoclamp 10.0, Molecular Devices, San Jose, CA, USA).

After vagus nerve positioning in the recording chamber, and with stimulation initiated and maintained, there was a stabilization period of 60 min of evoked CAP necessary to obtain a stable peak-to-peak amplitude of the signal. Following the stabilization period, two types of stimulus were used: a square wave pulse with 40 V in amplitude and 0.1 ms duration to evoke responses of myelinated fibers, and a square wave pulse with 60 V in amplitude and 1.5 ms duration to evoke responses of unmyelinated fibers. Both types of stimulus were supramaximal for the respective type of response desired. The frequency of tissue stimulation was set to 0.2 Hz in all cases.

The CAP parameters registered that were associated with conductibility were peak-to-peak amplitude, conduction velocity and duration at 50% of peak amplitude. The peak-to-peak amplitude was quantified by the sum of the greatest positive amplitude plus the absolute value of the most negative amplitude of the CAP signal. The conduction velocity of a given CAP component was measured by the ratio of the length of nerve trunk between stimulation and registering electrodes and the time interval between stimulus artifact and the peak of that CAP component. Regarding vagus nerve excitability, rheobase and chronaxie parameters were measured. The rheobase is assumed to be the lower stimulus pulse necessary to evoke a nerve response when it is stimulated by a very long pulse (1.0 ms for myelinated fibers and 3.0 ms for unmyelinated fibers). In turn, the chronaxie is the minimum time possible to evoke a nerve response when a pulse that is double that of the rheobase is applied to the nerve trunk.

### 4.4. Statistical Analysis

Data are expressed as mean ± standard error of the mean and “*n*” indicates the quantity of nerves used (1 nerve/animal). For parametric data, one-way ANOVA followed by appropriate comparison tests were used to compare animal groups versus control or between themselves. For non-parametric data, one-way ANOVA on ranks and appropriate post hoc tests were employed. The statistical difference between groups were considered significant when *p* < 0.05.

## 5. Patents

The data from this manuscript was part of a request for patent deposit, submitted to a patent base in Brazil (www.gov.br/inpi; accessed on 26 April 2021), which received the access number: BR 102018075807-1 A2, 2020.

## Data Availability

Not applicable.
